# Brassinosteroids control root epidermal cell fate via direct regulation of a MYB-bHLH-WD40 complex by GSK3-like kinases

**DOI:** 10.7554/eLife.02525

**Published:** 2014-04-25

**Authors:** Yinwei Cheng, Wenjiao Zhu, Yuxiao Chen, Shinsaku Ito, Tadao Asami, Xuelu Wang

**Affiliations:** 1 State Key Laboratory of Genetic Engineering, and Institute of Plant Biology, School of Life Sciences, Fudan University, Shanghai, China; 2 Graduate School of Agricultural and Life Sciences, The University of Tokyo, Tokyo, Japan; 3 College of Life Science and Technology, Huazhong Agricultural University, Wuhan, China; University of California, Berkeley and USDA Agricultural Research Service, United States

**Keywords:** brassinosteroids, GSK3-like kinases, root epidermal cell fate, EGL3, phosphorylation, TTG1, *Arabidopsis*

## Abstract

In *Arabidopsis*, root hair and non-hair cell fates are determined by a MYB-bHLH-WD40 transcriptional complex and are regulated by many internal and environmental cues. Brassinosteroids play important roles in regulating root hair specification by unknown mechanisms. Here, we systematically examined root hair phenotypes in brassinosteroid-related mutants, and found that brassinosteroid signaling inhibits root hair formation through GSK3-like kinases or upstream components. We found that with enhanced brassinosteroid signaling, *GL2*, a cell fate marker for non-hair cells, is ectopically expressed in hair cells, while its expression in non-hair cells is suppressed when brassinosteroid signaling is reduced. Genetic analysis demonstrated that brassinosteroid-regulated root epidermal cell patterning is dependent on the WER-GL3/EGL3-TTG1 transcriptional complex. One of the GSK3-like kinases, BIN2, interacted with and phosphorylated EGL3, and EGL3s mutated at phosphorylation sites were retained in hair cell nuclei. BIN2 phosphorylated TTG1 to inhibit the activity of the WER-GL3/EGL3-TTG1 complex. Thus, our study provides insights into the mechanism of brassinosteroid regulation of root hair patterning.

**DOI:** http://dx.doi.org/10.7554/eLife.02525.001

## eLife digest

Roots anchor a plant into the ground, and allow the plant to absorb water and mineral nutrients from the soil. As roots grow and branch, they increase the surface area of root exposed to the soil—and many plant cells in the root's outer layer have a hair-like projection to further increase this surface area. Thus, root hairs are where most water and mineral nutrients are absorbed. Many factors affect whether, or not, a plant cell will develop into a root hair. These factors include both external cues (such as the mineral content of the soil) and signals from the plant itself (such as hormones).

Brassinosteroids are plant hormones that regulate the development of shoots and roots, as well as the timing of when flowers begin to develop. These hormones are detected on the outside of plant cells, and activate a signaling pathway within the cell that causes changes in gene expression. Brassinosteroids also control if a root cell will become a hair cell or not, although the mechanism behind this activity is unclear.

Here, Cheng et al. have looked at the root hairs of mutant *Arabidopsis thaliana* plants that have had individual genes involved in brassinosteroid signaling knocked-out. Plant biologists commonly study this plant species because it is small and grows quickly—and *Arabidopsis* has regular stripes of root hair cells and ‘non-hair cells’ in the outer layer of its roots. Cheng et al. reveal that brassinosteroids prevent the formation of root hairs via signaling pathways that involve proteins called GSK3-like kinases. These hormones ‘switch off’ these kinases’ activity, so knocking-out the genes that code for these kinases has the same effect as adding extra brassinosteroids to the plant roots: fewer root hair cells.

Cheng et al. show that one of the GSK3-like kinases binds and adds phosphate groups to protein complexes that control gene expression—and this causes these protein complexes to be less active. When GSK3-like kinase activity is switched off by brassinosteroids, these complexes instead become more active and trigger the expression of genes that direct a plant cell to become a non-hair cell.

The findings of Cheng et al. reveal the pathways that allow brassinosteroids to stop plant cells in roots from becoming hair cells, and that instead encourage these cells to become non-hair cells. However, further work is needed to uncover how the striped pattern of hair cells and non-hair cells on *Arabidopsis* roots is established, and how brassinosteroids work with other plant hormones to control this pattern.

**DOI:** http://dx.doi.org/10.7554/eLife.02525.002

## Introduction

The *Arabidopsis* root epidermal cell types are defined by position in a predictable manner (Ishida et al., 2008). Hair (H) cells, or trichoblasts, are specified early from root epidermal cells that lie over clefts between two underlying cortical cells, whereas the root epidermal cells that lie over a single cortical cell develop as non-hair (N) cells, or atrichoblasts (Ishida et al., 2008). Hair cell and non-hair cell files are patterned alternately in rows within the *Arabidopsis* root epidermis, with columns of hair cells interspersed with columns of non-hair cells (Schiefelbein et al., 2009). Prior to root hair outgrowth, root epidermal cells in the H position can be distinguished from those in the N position by many visible cellular features, including a greater rate of cell division (Berger et al., 1998), reduced cell length and vacuolation (Dolan et al., 1994; Galway et al., 1994), and enhanced cytoplasmic density (Dolan et al., 1994). It is proposed that positional signals and a putative receptor-like kinase SCRAMBLED (SCM) (Kwak et al., 2005) function through a MYB-bHLH-WD40 repeat transcriptional complex to determine root epidermal cell fate (Schiefelbein et al., 2009). Based on this model, in N cells, WEREWOLF (WER) (Lee and Schiefelbein, 1999), a R2R3 MYB-domain transcription factor, forms a complex with basic helix-loop-helix transcription factors, GLABRA3 (GL3)/ENHANCER OF GLABRA3 (EGL3) (Bernhardt et al., 2003; Zhang et al., 2003), and a WD40 repeat protein, TRANSPARENT TESTA GLABRA1 (TTG1) (Galway et al., 1994), to promote expression of *GLABRA2* (*GL2*) and *CAPRICE* (*CPC*) (Ryu et al., 2005; Song et al., 2011). GL2, a homeodomain/leucine zipper transcription factor, negatively regulates H cell fate and positively regulates N cell fate (Masucci et al., 1996). CPC (Wada et al., 1997), a MYB-type transcription factor, moves from N cells to H cells (Kurata et al., 2005a) to compete with WER for binding to GL3/EGL3 to form a CPC-GL3/EGL3-TTG1 complex, which is unable to induce *GL2* expression (Song et al., 2011). In addition to CPC, the bHLH transcription factor GL3 is also a mobile protein (Bernhardt et al., 2005). *GL3* and its homologue *EGL3* are both expressed in H cells, but GL3 protein is only localized in the N cell nucleus, indicating that GL3 protein moves into the adjoining N cell nucleus to determine N cell fate (Bernhardt et al., 2003, 2005). Integration of existing genetic and biochemical data also supports an alternative mechanism centered on the movement of transcriptional factors between epidermal cells rather than a putative local activation of the *WER* gene function to determine root epidermis pattern formation (Savage et al., 2008).

In addition, root hair development is highly regulated by many external and internal cues, including phytohormones. For instance, abscisic acid (ABA) plays a role in the early stage of root epidermal cell specification (Van Hengel et al., 2004) and in inhibiting root hair tip growth in *Arabidopsis* (Schnall and Quatrano, 1992), while both ethylene and auxin may act downstream of TTG1 and GL2 to promote root hair formation and elongation (Masucci and Schiefelbein, 1994, 1996). Moreover, jasmonic acids (JAs) promote root hair formation through their interaction with ethylene (Zhu et al., 2006). However, the underlying cellular and molecular mechanisms of how these internal hormones integrate with environmental cues to regulate root hair cell fate determination are still poorly understood.

The plant steroid hormones, brassinosteroids (BRs), play essential roles in regulating many developmental processes, including shoot, root, and reproductive development (Savaldi-Goldstein et al., 2007; Ye et al., 2010; Hacham et al., 2011; Yang et al., 2011). BRs are perceived by the receptor kinase BRASSINOSTEROID INSENSITIVE 1 (BRI1) (Li and Chory, 1997; Hothorn et al., 2011; She et al., 2011). The BR-activated BRI1 phosphorylates BRI1 KINASE INHIBITOR 1 (BKI1) to release its inhibition (Wang and Chory, 2006), and then BKI1 acts as a positive regulator by binding to a subset of 14-3-3 proteins (Wang et al., 2011). Another BRI1 substrate, BR-SIGNALING KINASE (BSK), transduces the BR signaling through *bri1* SUPPRESSORS 1 (BSU1) to inactivate a GSK3-like kinase BRASSINOSTEROID INSENSITIVE 2 (BIN2), which leads to accumulation of the dephosphorylated form of transcriptional factors BRI1 EMS SUPPRESSOR 1 (BES1)/BRASSINAZOLE RESISTANT 1 (BZR1) in the nucleus to regulate gene expression (Yang et al., 2011). A previous study suggests that BRs play an important role in determining root epidermal cell fate by regulating *WER* and *GL2* expression (Kuppusamy et al., 2009). However, the elaborate molecular mechanism by which BRs regulate root epidermal cell fate and development is still unknown.

Here, we first systematically examined root epidermal cell patterning and *PGL2::GUS* expression in a series of BR-deficient and signaling mutants. We found that BRs regulate root epidermal cell fate through promoting *GL2* expression in both H and N cells, which is mediated by GSK3-like kinases and the WER-GL3/EGL3-TTG1 complex as indicated by genetic analysis and biochemical studies. Our study further demonstrated that BIN2, one of the GSK3-like kinases, interacted with and phosphorylated EGL3 on T399 and T209/T213, leading to its trafficking from nucleus to cytosol in H cells, which may facilitate its movement from H cells to N cells. BIN2 also phosphorylated TTG1 to inhibit the activity of the WER-GL3/EGL3-TTG1 transcriptional complex. These results explain how BR signaling regulates both the formation and activity of the WER-GL3/EGL3-TTG1 complex through GSK3-like kinases to coordinate root epidermal cell fate specification.

## Results

### BRs regulate root epidermal cell patterning through GSK3-like kinases

**Figure 1.**
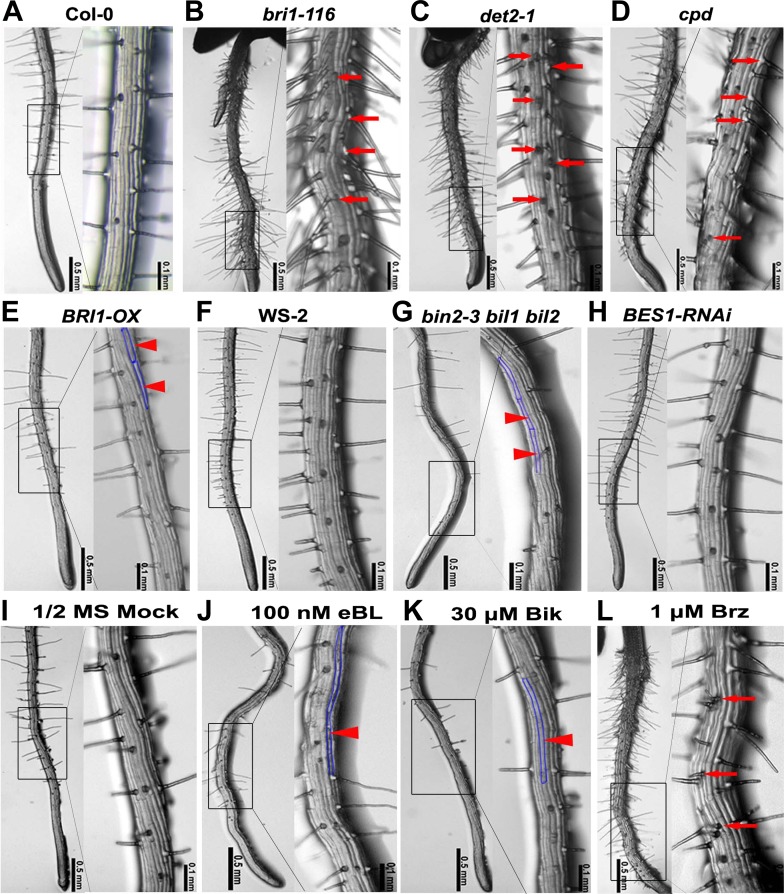
Root epidermal cell patterning is altered in the BR-related mutants. (**A**–**H**) Root hair patterning of the BR-related mutants and their wild type counterparts. *bin2-3 bil1 bil2* is in the WS-2 background, and all of the others are in the Col-0 background. (**I**–**L**) Root hair phenotype of the wild type plants grown on 1/2 MS (Murashige and Skoog) medium with DMSO (mock) (**I**), 100 nM epibrassinolide (eBL) (**J**), 30 μM bikinin (Bik) (**K**), or 1 μM brassinazole (Brz) (**L**). Right images are the outlined areas of left images with higher magnification. Arrows indicate ectopic root hair cells, and arrowheads indicate ectopic non-hair cells. Areas outlined with blue lines indicate the ectopic non-hair cells. **DOI:** http://dx.doi.org/10.7554/eLife.02525.003 10.7554/eLife.02525.004Figure 1—source data 1.Root hair density, cell length, and relative hair number of the BR-related mutants and wild type plants treated with eBL, bikinin, Brz, or DMSO**DOI:** http://dx.doi.org/10.7554/eLife.02525.004

To broadly explore the role of BRs in root hair formation, we systematically examined the root hair phenotype of BR-biosynthetic mutants, *det2-1* and *cpd*, and BR-responsive mutants, including *bri1-116*, *BRI1-OX* (a *BRI1*-overexpression line), *bin2-3 bil1 bil2* (a triple knockout mutant of *BIN2* and its two closest homologues), and a *BES1-RNAi* line. We found that the relative hair number (=root hair density × root hair cell length) was higher in *bri1-116* (4.67 ± 0.47), *det2-1* (4.75 ± 0.52), and *cpd* (4.65 ± 0.54), and significantly lower in *BRI1-OX* (3.40 ± 0.46) and *bin2-3 bil1 bil2* (2.86 ± 0.39) than in their corresponding wild types Col-0 (3.89 ± 0.43) and WS-2 (3.85 ± 0.41) (Figure 1—source data 1). However, there was no significant difference between *BES1-RNAi* and Col-0. Images at the highest magnification (×100; Figure 1) showed that in the wild type root (Figure 1A), the H cells and N cells were arranged in alternating files with the H cell columns regularly interspaced with the N cell columns; no adjacent H cell columns were found. However, in the BR signaling-inhibited mutants, including *bri1-116*, *det2-1*, and *cpd* (Figure 1B–D), many root hair columns were next to each other, leading to more root hairs, suggesting that some N cell fate might be changed into H cell fate. In contrast, the BR signaling-enhanced plants, including *BRI1-OX* and *bin2-3 bil1 bil2*, grew fewer root hairs than the wild type, due to the fact that they lacked root hairs in many H cell positions (Figure 1A,E–G). Interestingly, the *BES1-RNAi* line showed a similar root hair pattern as the wild type (Figure 1A,H).

**Figure 1—figure supplement 1.**
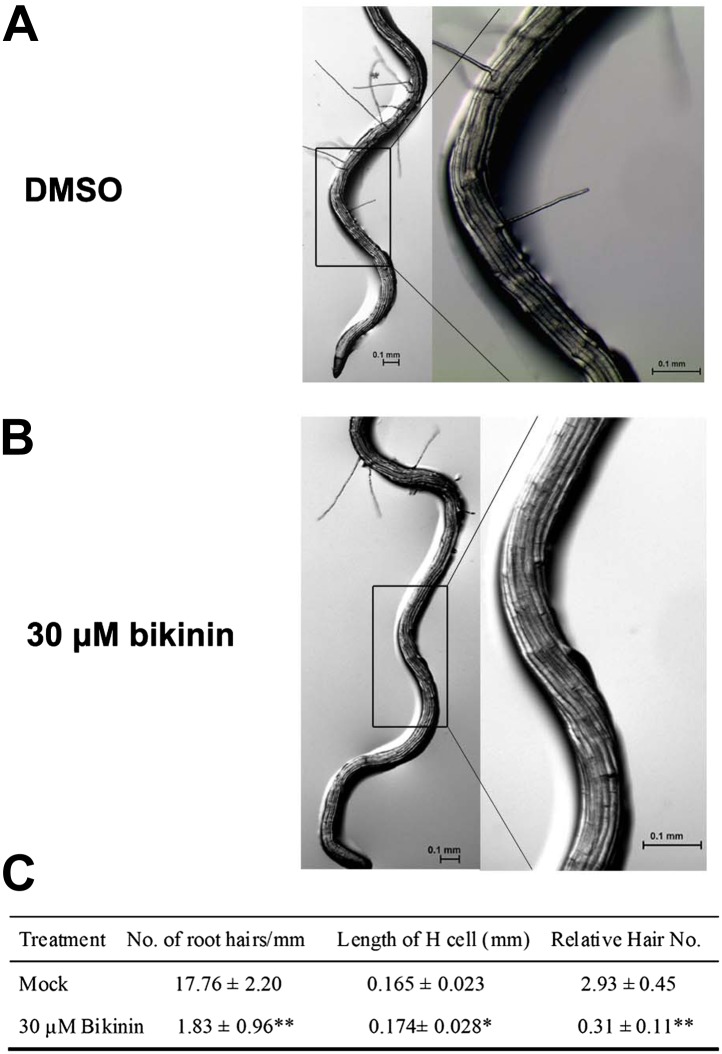
Bikinin treatment inhibited H cell fate in *bin2-3 bil1 bil2* mutants. Root hair phenotype of the *bin2-3 bil1 bil2* mutants grown on medium with DMSO (mock) (**A**) or 30 μM bikinin (**B**); right images are the outlined areas of left images with higher magnification. (**C**) The root hair density, cell length, and relative hair number of the *bin2-3 bil1 bil2* mutants treated with bikinin. Values are means ± SD. The two-tailed *t* test with equal variance or unequal variance was used to determine the significance level of the difference between the *bin2-3 bil1 bil2* mutants treated with 30 μM bikinin and mock medium. *p<0.05; **p<0.01. **DOI:** http://dx.doi.org/10.7554/eLife.02525.005

To further test whether exogenously applied eBL (epibrassinolide), bikinin (a specific GSK3 kinase inhibitor) (De Rybel et al., 2009), or Brz (brassinazole, an inhibitor of BR biosynthesis) regulate root hair specification, we planted seeds on 1/2 MS (Murashige and Skoog) medium containing each of these chemicals or DMSO (as the mock treatment), and carefully observed their root hair phenotypes. We found that compared with plants grown on the mock medium (3.80 ± 0.45), the relative root hair number of plants grown on medium containing 100 nM eBL (3.22 ± 0.42) or 30 μM bikinin (2.71 ± 0.50) was significantly reduced, while it was greatly increased in plants grown on medium containing 1 μM Brz (4.17 ± 0.43) (Figure 1—source data 1). Images at higher magnification (×100) show that, compared to seedlings grown on the mock medium (Figure 1I), those grown on medium containing eBL or bikinin produced fewer root hairs in the H position of epidermal cells (Figure 1J,K), while those grown on medium containing Brz grew more root hairs in the N position (Figure 1L). We also found that the *bin2-3 bil1 bil2* seedlings grown on medium containing 30 μM bikinin produced very few root hairs (Figure 1—figure supplement 1), suggesting that besides *BIN2*, *BIL1*, and *BIL2*, other GSK3-like kinases may also be involved in root hair specification in *Arabidopsis*. Taken together, these findings indicate that the BR-mediated root epidermal cell pattern formation largely relies on GSK3-like kinases and/or their upstream components.

### BR signaling promotes N cell fate and inhibits H cell fate

**Figure 2.**
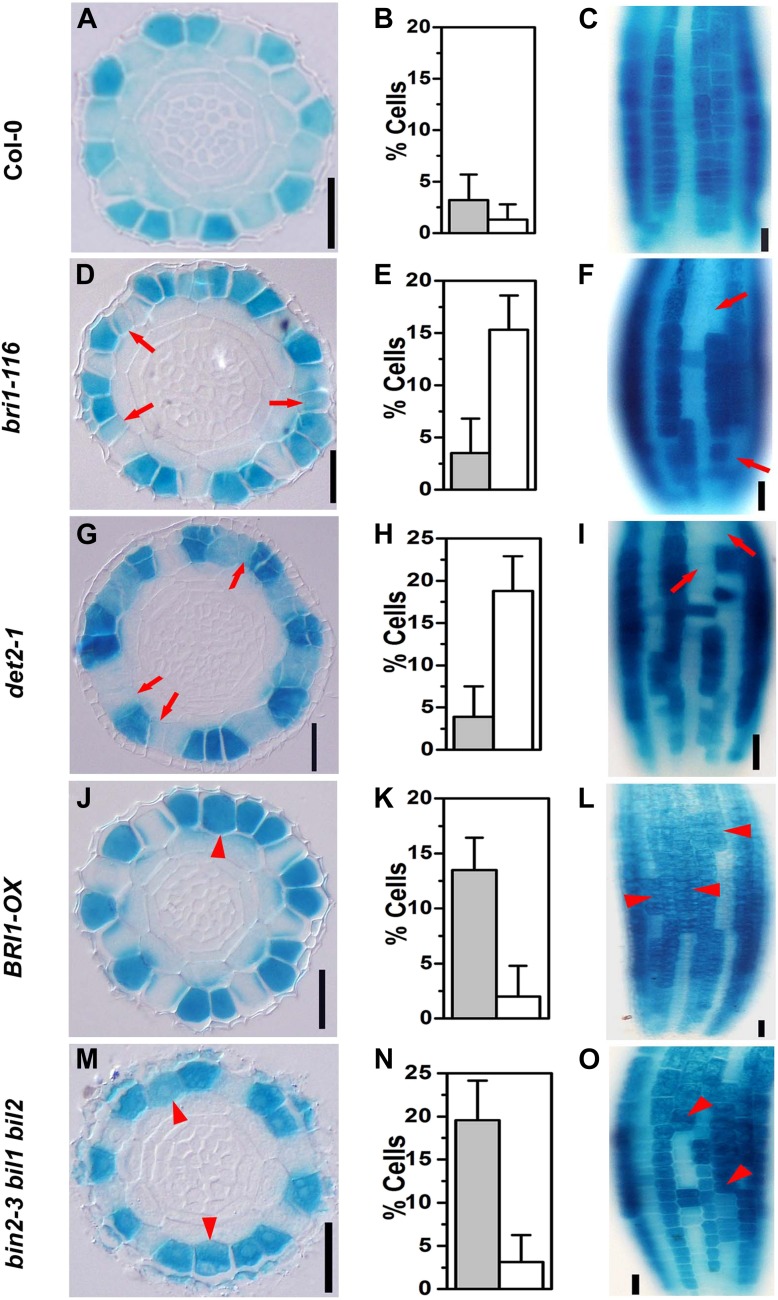
Expression pattern of *PGL2::GUS* is altered in the BR-related mutants. Transverse sections from root meristem of Col-0 (**A**), *bri1-116* (**D**), *det2-1* (**G**), *BRI1-OX* (**J**), and *bin2-3 bil1 bil2* (**M**). Frequency of cells without *PGL2::GUS* expression in the N cell position (open bars) and cells with ectopically expressed *PGL2::GUS* in the H cell position (solid bars) of Col-0 (**B**), *bri1-116* (**E**), *det2-1* (**H**), *BRI1-OX* (**K**), and *bin2-3 bil1 bil2* (**N**). Longitudinal images of the root epidermal cells in Col-0 (**C**), *bri1-116* (**F**), *det2-1* (**I**), *BRI1-OX* (**L**), and *bin2-3 bil1 bil2* (**O**). Scale bars, 25 μm. Red arrows indicate N cells without *PGL2::GUS* expression, and red arrowheads indicate H cells ectopically expressing *PGL2::GUS*. For each genotype, n = 8. Error bars indicate standard deviation (SD). **DOI:** http://dx.doi.org/10.7554/eLife.02525.006

*GL2* has been widely used as a molecular maker of N cell fate determination (Masucci et al., 1996; Kuppusamy et al., 2009). In order to test whether the disordered root hair patterning in the BR-related mutants resulted from an altered root epidermal cell fate, we analyzed the *GL2* expression pattern in these mutants using *PGL2::GUS* as a reporter. We found that in the wild type, 1.3% of epidermal cells in the N position lacked *GL2* expression in cross-sections (Figure 2A,B), and in the longitudinal view of root epidermis, the root epidermal cell files were arranged regularly, with *GL2*-expressing columns (N cell columns) interspaced with columns without *GL2* expression (H cell columns) (Figure 2C). However, about 15.3% of *bri1-116* and 18.8% of *det2-1* cells showed suppressed *PGL2::GUS* expression, and there were adjacent root epidermal cells without *GL2* expression in both *bri1-116* and *det2-1* (Figure 2D–I), which supports a previous finding with *bri1-116* and the Brz-treated wild type (Kuppusamy et al., 2009). These results indicated that adjacent root hairs in *bri1-116* and *det2-1* were caused by some N cell fate changing to H cell fate. In contrast, *GL2* was ectopically expressed in about 13.5% of H cells in *BRI1-OX* plants (Figure 2J–L) and in about 19.6% in *bin2-3 bil1 bil2* (Figure 2M–O), as compared with only 3.2% in the wild type (Figure 2A–C), indicating that lack of *GL2* expression in N cells may correspond to the ectopic root hairs observed in *bri1-116* and *det2-1*, and that the ectopically expressed *GL2* in H cells partially inhibits H cell fate in *BRI1-OX* and *bin2-3 bil1 bil2* plants. Taken together, the above results suggested that BR signaling has an important role in suppressing H cell fate and promoting N cell fate in both the N and the H positions, and BR signaling regulates root epidermal cell fate by controlling *GL2* expression through GSK3-like kinases, or their upstream components, but not through downstream transcription factors.

### BR signaling acts upstream of WER and CPC to regulate root epidermal cell fate

**Figure 3.**
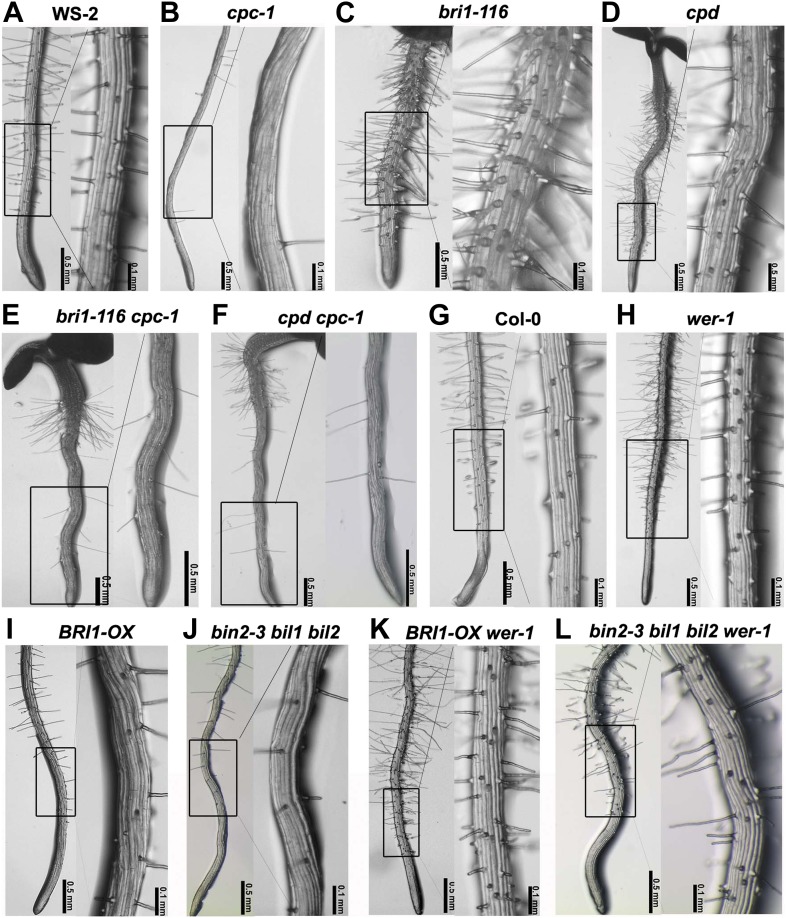
BR signaling acts upstream of CPC and WER to regulate root epidermal cell fate. Root hair phenotype of the wild type WS-2 (**A**) and double mutants of *cpc-1* (**B**) with *bri1-116* (**C**) or *cpd* (**D**), including *bri1-116 cpc-1* (**E**) and *cpd cpc-1* (**F**). Root hair phenotype of the wild type Col-0 (**G**) and the double/multiple mutants of *wer-1* (**H**) with *BRI1-OX* (**I**) or *bin2-3 bil1 bil2* (**J**), including *BRI1-OX wer-1* (**K**) and *bin2-3 bil1 bil2 wer-1* (**L**). *cpc-1* is in the WS-2 background. BR: brassinosteroid. **DOI:** http://dx.doi.org/10.7554/eLife.02525.007

It was known that *GL2* expression is directly regulated by the WER-GL3/EGL3-TTG1 but not the CPC-GL3/EGL3-TTG1 transcriptional complex (Schiefelbein et al., 2009). To explore whether the BR-regulated *GL2* expression and root epidermal cell fate determination are dependent on these complexes, we first created a set of double mutants of *cpc-1*, a mutant with fewer root hairs than its counterpart (Figure 3A,B), with *bri1-116* or *cpd* (Figure 3C,D). Similar to *cpc-1*, both double mutants *bri1-116 cpc-1* (Figure 3E) and *cpd cpc-1* (Figure 3F) produced few root hairs. We also generated double or multiple mutants of *wer-1*, a mutant with more root hairs than Col-0 (Figure 3G,H), with *BRI1-OX* or *bin2-3 bil1 bil2* (Figure 3I,J), and found that both *BRI1-OX wer-1* and *bin2-3 bil1 bil2 wer-1* (Figure 3K,L) were similar to *wer-1*, with many ectopic root hairs formed at the N cell position. These genetic analyses indicated that the WER-GL3/EGL3-TTG1 and CPC-GL3/EGL3-TTG1 transcriptional complexes act downstream of BR early signaling.

### BIN2 phosphorylates EGL3 and TTG1

**Figure 4.**
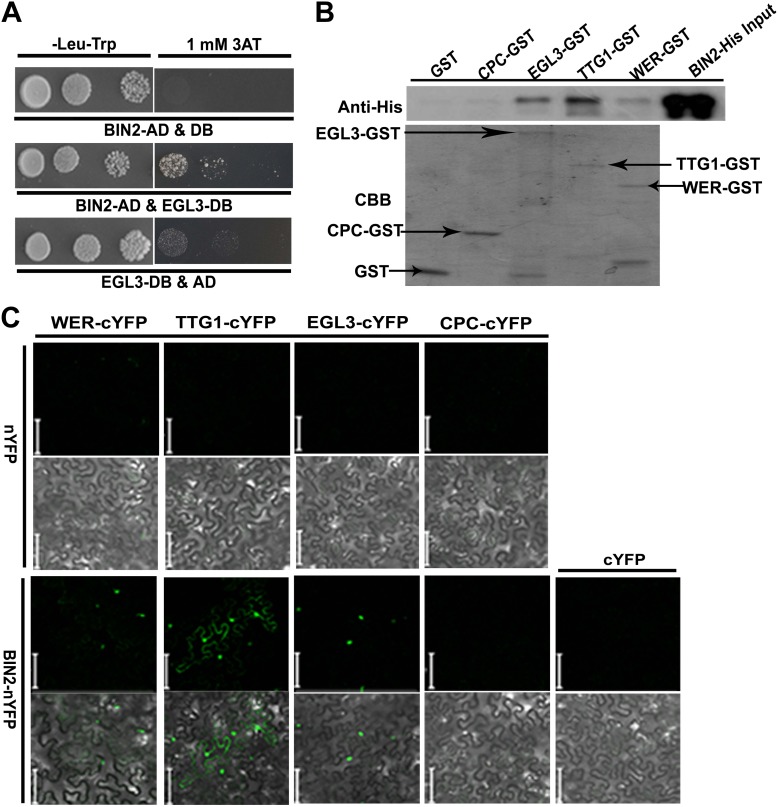
BIN2 interacts with EGL3, TTG1, and WER. (**A**) BIN2 interacts with EGL3 in yeast two-hybrid assays. (**B**) The interaction of BIN2-His with CPC-GST, EGL3-GST, TTG1-GST, and WER-GST in vitro. The BIN2-His pulled-down by CPC-GST, EGL3-GST, TTG1-GST, and WER-GST, or GST was detected by western blotting with anti-His antibody (top). The purified BIN2-His protein was used as inputs. An equal loading of recombinant proteins was indicated by Coomassie brilliant blue (CBB) staining (bottom). (**C**) BiFC assays of the interaction between BIN2 with EGL3, TTG1, and WER. Scale bars, 20 μm. **DOI:** http://dx.doi.org/10.7554/eLife.02525.008

**Figure 5.**
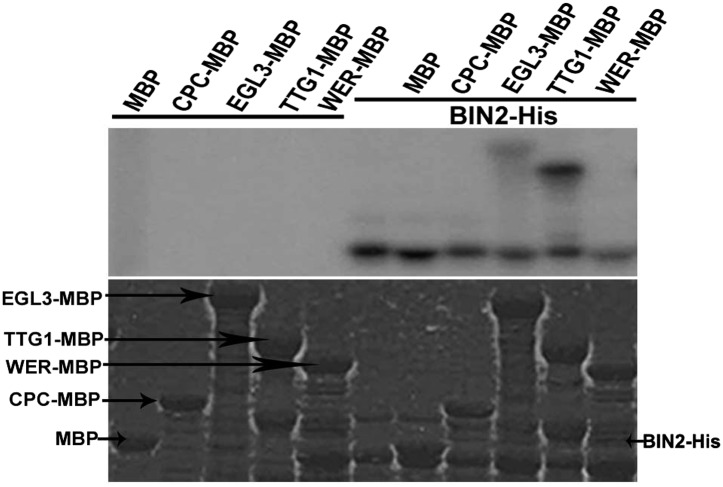
BIN2 phosphorylates EGL3 and TTG1, but not WER and CPC in vitro. An equal amount of recombinant BIN2 kinase indicated by Coomassie brilliant blue (CBB) staining (bottom) was incubated with recombinant MBP, WER-MBP, CPC-MBP, EGL3-MBP, or TTG1-MBP, separated by SDS–PAGE, and followed by autoradiography (top). **DOI:** http://dx.doi.org/10.7554/eLife.02525.010

Therefore, we inferred that BR-mediated root epidermal cell fate may be dependent on GSK3-like kinases, the key negative components in the BR signaling pathway, acting upstream of the WER-EGL3/GL3-TTG1 or CPC-EGL3/GL3-TTG1 transcriptional complex. We then conducted yeast two-hybrid assays to test whether any components in the WER-EGL3/GL3-TTG1 or CPC-EGL3/GL3-TTG1 complex interact with BIN2, a well-studied GSK3-like kinase, and found that BIN2 can interact with EGL3 (Figure 4A) but not with CPC in yeast (Figure 4—figure supplement 1). However, due to strong auto-activation of WER and TTG1 fused with GAL4-DNA binding domain (DB) in yeast two-hybrid assays (Figure 4—figure supplement 1), we conducted GST pull-down and BiFC (biomolecular fluorescence complementation) assays to test their interactions, and found that BIN2 can interact with WER, EGL3, and TTG1 (Figure 4B,C). Furthermore, because BIN2 can regulate many transcription factors by phosphorylation (Saidi et al., 2012), and WER or CPC can interact with EGL3/GL3-TTG1 in vivo to form WER-EGL3/GL3-TTG1 or CPC-EGL3/GL3-TTG1 complexes, respectively (Zhao et al., 2008; Song et al., 2011), we then conducted in vitro kinase assays to test whether BIN2 can phosphorylate any of these components. We found that BIN2 did not phosphorylate WER and CPC, but was able to phosphorylate EGL3 and TTG1 (Figure 5).

**Figure 4—figure supplement 1.**
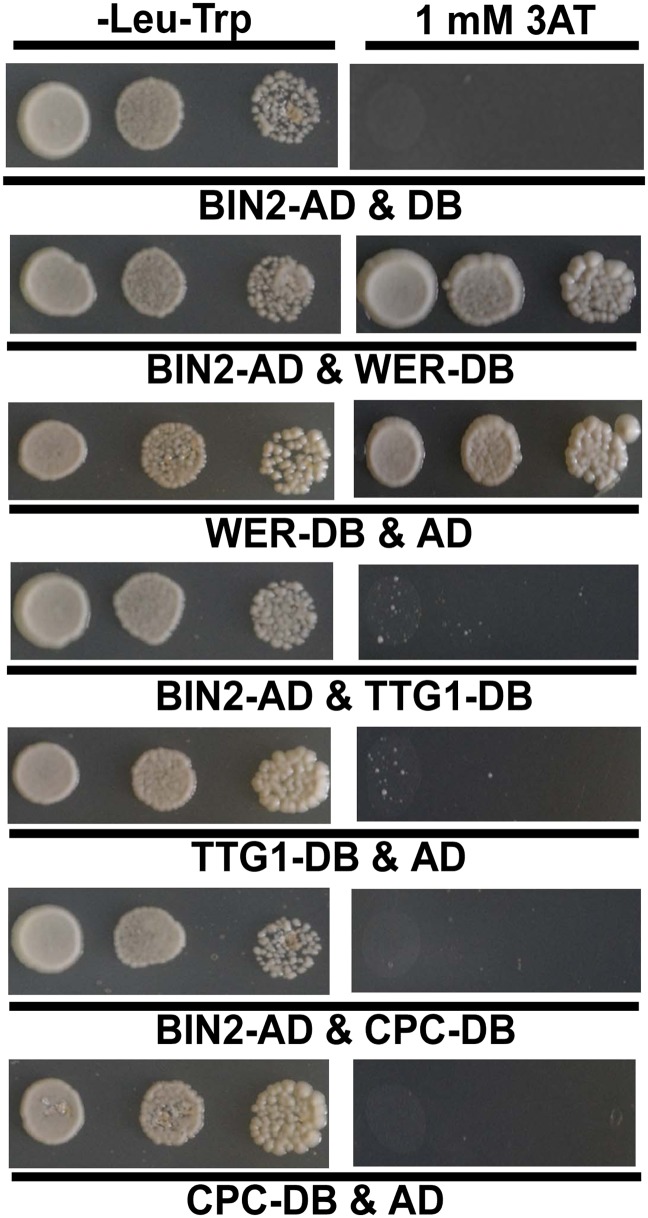
Yeast two-hybrid assays to test interactions of BIN2 with WER, TTG1, or CPC. The full-length cDNA of each corresponding gene was fused with the GAL4-BD or AD domain. Yeast cells harboring the indicated constructs were grown on the synthetic media lacking Trp and Leu, or Trp, Leu, and His with an additional 1 mM 3AT. **DOI:** http://dx.doi.org/10.7554/eLife.02525.009

**Figure 5—figure supplement 1.**
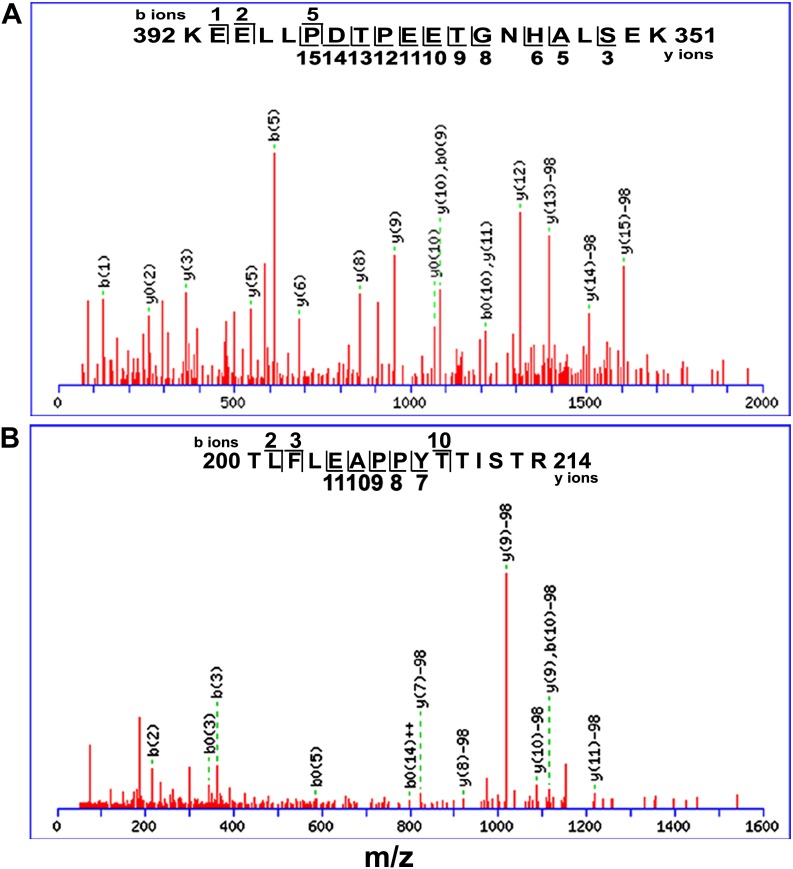
Mass spectrometry analysis of EGL3 phosphorylation sites. Putative phosphorylation sites are in T^399^PEET^403^ (**A**) and T^209^TIST^213^ fragments (**B**). **DOI:** http://dx.doi.org/10.7554/eLife.02525.011

### GSK3-like kinase phosphorylates EGL3 to promote its movement to N cells

**Figure 6.**
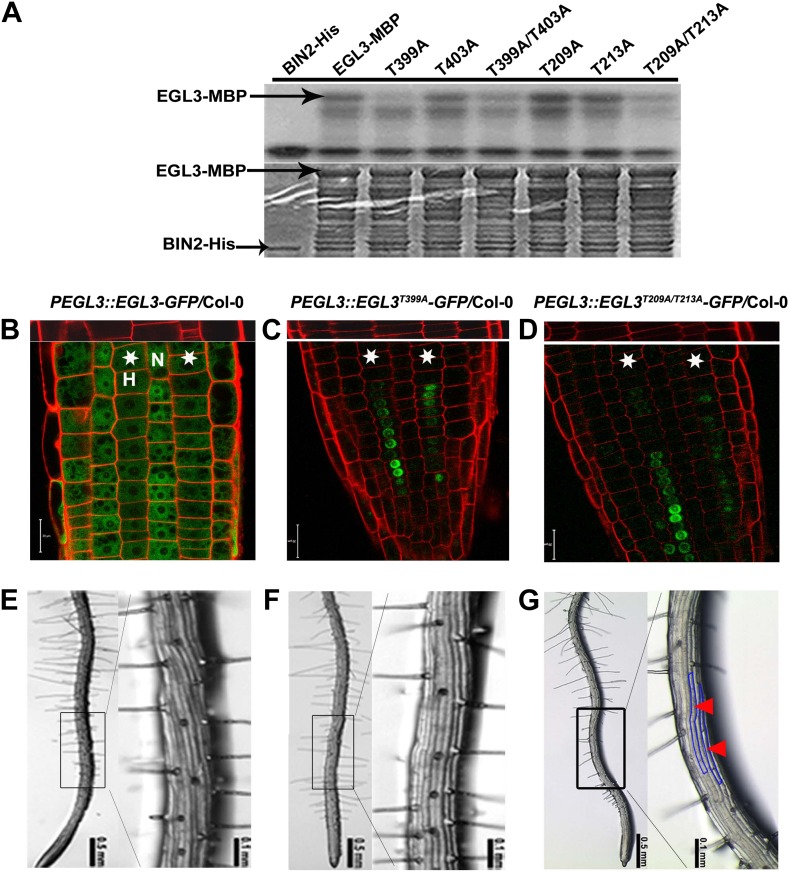
BIN2 phosphorylates EGL3 to regulate its subcellular localization and root epidermal cell fate. (**A**) BIN2 phosphorylates EGL3 on T399 and T209/T213. An equal amount of recombinant protein, as indicated by Coomassie brilliant blue (CBB) staining (bottom panel), was incubated in phosphorylation buffer, separated by SDS–PAGE, and followed by autoradiography (top panel). (**B**) EGL3-GFP is predominantly localized in N cell nuclei. Both EGL3^T399A^-GFP (**C**) and EGL3^T209A/T213A^-GFP (**D**) are solely localized in H cell nuclei. For (**B**–**D**), the 5-day-old roots were stained with propidium iodide (red) for 10 s for visualizing the cell wall. The top panels show the underlying cortex. The stars indicate H cells. Scale bars, 20 μm. (**E**–**G**) Root hair patterns of *EGL3-GFP* (**E**), *EGL3*^*T399A*^*-GFP* (**F**), and *EGL3*^*T209A/T213A*^*-GFP* (**G**) transgenic plants. Outlined areas in the left images are magnified in the right images. Red arrowheads and areas outlined with blue lines indicate ectopic non-root hair cells in the H position. **DOI:** http://dx.doi.org/10.7554/eLife.02525.012 10.7554/eLife.02525.013Figure 6—source data 1.EGL3 amino acid sequence analysis**DOI:** http://dx.doi.org/10.7554/eLife.02525.013

To investigate the biological relevance of EGL3 phosphorylation by a GSK3-like kinase BIN2, we used mass spectrometry and identified four potential phosphorylation sites (T209, T213, T399, and T403) of EGL3 by BIN2 (Figure 5—figure supplement 1), which are located in two regions that contain typical recognition sites of GSK3 kinases (Cohen and Frame, 2001). We then mutated threonine residues into alanine to make single- or double-site mutated forms of EGL3. In vitro phosphorylation assays showed that phosphorylation levels of EGL3^T399A^ and EGL3^T209A/T213A^ by BIN2 were significantly reduced (Figure 6A), indicating that T399 and T209/T213 are the main phosphorylated residues. To investigate the biological function of EGL3 phosphorylation, we transformed *EGL3* and its mutated forms driven by its own promoter into Col-0 to examine their subcellular localization. We found that the EGL3-GFP signal in N cells was apparently higher than that in H cells, and that it was mainly localized to cytosol in H cells, but to both cytosol and nucleus in N cells (Figure 6B). As *EGL3* mRNA is only expressed in H cells (Bernhardt et al., 2005), this result indicated that, like its homologue GL3, EGL3 is also a mobile protein that moves from H cells to N cells. However, the EGL3^T399A^-GFP and EGL3^T209A/T213A^-GFP were solely localized to the nuclei of H cells (Figure 6C,D), indicating that EGL3 phosphorylation is required not only for its cytoplasmic accumulation, but also for its movement from H cells to N cells. In addition, we found that the root hair patterning of *EGL3-GFP* transgenic plants was not altered (Figure 6E; Table 1), indicating that correctly localized EGL3, mainly in N cell nuclei to promote N cell fate and less in H cell nuclei not to promote N cell fate, has no influence on root epidermal fate. Moreover, although root epidermal patterning in the *EGL3*^*T399A*^*-GFP* transgenic plants was normal (Figure 6F; Table 1), the number of root hairs in *EGL3*^*T209A/T213A*^*-GFP* plants was significantly reduced, likely due to a misspecification of non-hair cells in the H position (Figure 6G; Table 1), suggesting that the nucleus-localized EGL3 in H cells may determine N cell fate specification. Taken together, our results indicate that BIN2 phosphorylation on T399, T209, and/or T213 of EGL3 in H cells promotes EGL3 cytoplasmic localization, which likely helps its movement from H to N cells to regulate root epidermal cell fate.

**Figure 6—figure supplement 1.**
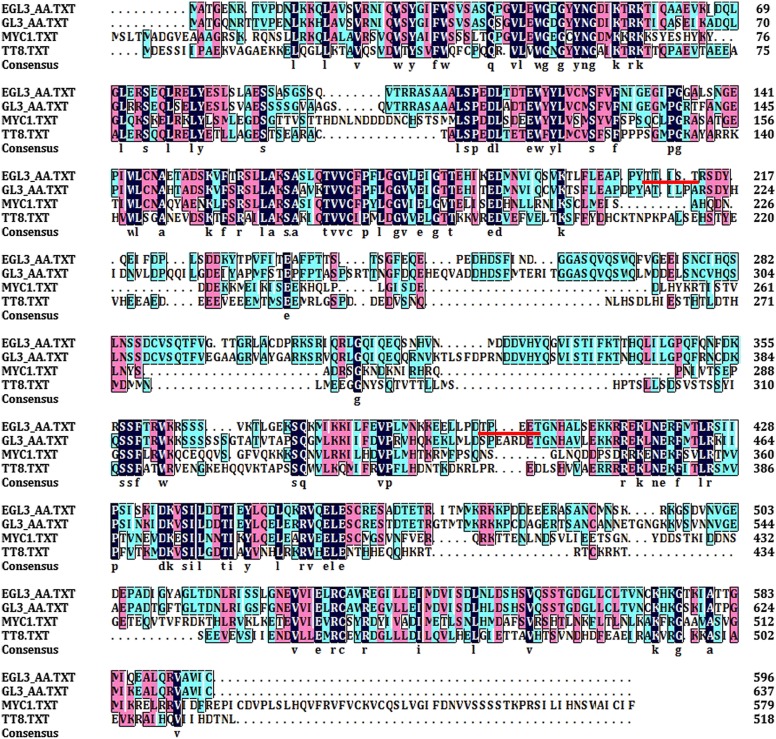
Alignment of EGL3 amino acid sequence with other bHLH homologues in *Arabidopsis*. The sequences underlined in red indicate the 209TTIST213 and 399TPEET403 regions of EGL3. **DOI:** http://dx.doi.org/10.7554/eLife.02525.014

**Table 1.** The effect of EGL3 and its two mutated forms on root epidermal cell pattern formation **DOI:** http://dx.doi.org/10.7554/eLife.02525.015

Genotype	Cells in the H position		Cells in the N position	
	Hair cells (%)	Non-hair cells (%)	Hair cells (%)	Non-hair cells (%)
Col-0	98.9 ± 3.3	1.1 ± 3.3	2.0 ± 4.5	98.0 ± 4.5
*PEGL3::EGL3-GFP*	95.8 ± 6.1	4.2 ± 6.1	1.9 ± 4.2	98.1 ± 4.2
*PEGL3::EGL3*^*T399A*^*-GFP*	93.1 ± 6.4	6.9 ± 6.4	2.8 ± 6.0	97.2 ± 6.0
*PEGL3::EGL3*^*T209A/T213A*^*-GFP*	85.5 ± 4.4*	14.5 ± 4.4*	1.0 ± 3.2	99.0 ± 3.2

At least 10 different 5-day-old roots were examined for each strain. Values represent means ± SD. For statistical analysis, the F test was used to determine the variance, and the two-tailed *t* test with equal variance or unequal variance was used to determine the significance level of the difference among the transgenic plants.

* p<0.05.

### GSK3-like kinases phosphorylate TTG1 to suppress the transcriptional activity of the WER-EGL3-TTG1 complex

**Figure 7.**
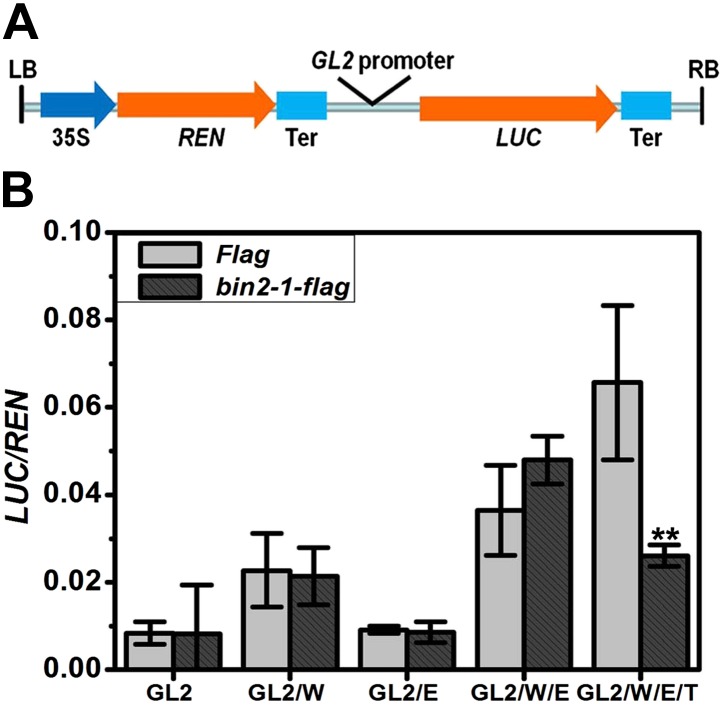
BIN2 inhibits the transcription activity of the WER-EGL3-TTG1 complex through TTG1. (**A**) Schematic diagram of the dual-luciferase reporter construct. The firefly luciferase (*LUC*) reporter gene was driven by *GL2* promoter. The Renillia luciferase (*REN*) reporter gene was controlled by Cauliflower mosaic virus promoter (35S) and terminator (Ter). (**B**) bin2-1 inhibits *PGL2::LUC* expression only when *TTG1* is co-expressed with *WER* and *EGL3*. Relative reporter activity in *Nicotiana benthamiana* leaf cells transiently transformed with the indicated effector, reporter, and regulatory constructs. G, W, E, and T indicate *GL2*, *WER*, *EGL3*, and *TTG1*, respectively. Error bars indicate SD. **p<0.01 determined by the two-tailed Student's *t* test. **DOI:** http://dx.doi.org/10.7554/eLife.02525.016

TTG1 is required for a normal expression level of *GL2* (Di Cristina et al., 1996) but not for its expression pattern (Hung et al., 1998). Transgenic *TTG1-GFP* plants driven by its own promoter indicated that TTG1 was preferentially localized in the cytoplasm and slightly in the nucleus of both N and H cells (Figure 7—figure supplement 1), which was consistent with the subcellular localization of its petunia homologue AN11 (De Vetten et al., 1997). To understand the biological relevance of TTG1 phosphorylation by a GSK3-like kinase, we conducted transient transcription assays in *Nicotiana benthamiana* leaves to examine whether BIN2 regulated the complex's activity in a TTG1-dependent manner. We constructed a dual-luciferase reporter system using *PGL2::LUC* as a reporter gene and *35S::REN* as an internal control (Figure 7A). Because the protein of the gain-of-function mutation bin2-1 (E263K) is more stable and has higher activity than the wild type BIN2 (Peng et al., 2008), and GSK3-like kinases are quite conserved among different species (Saidi et al., 2012), we used bin2-1 to conduct this study. As shown in Figure 7B, transient expression of *WER* alone was able to slightly induce *PGL2::LUC* gene expression. In contrast, transient expression of *EGL3* alone was unable to induce reporter gene expression. Co-expression of *WER* and *EGL3* can dramatically promote *LUC* expression, which is consistent with a previous study (Song et al., 2011). Additional bin2-1 did not alter the effect of WER, EGL3, or both WER and EGL3 on *PGL2::LUC* expression. When WER, EGL3, and TTG1 were used together, the expression of *PGL2::LUC* was further enhanced, indicating that TTG1 can promote the activity of the WER-EGL3 complex. Interestingly, additional bin2-1 significantly inhibited reporter gene expression regulated by the WER-EGL3-TTG1 complex (Figure 7B), indicating that TTG1 is mediating the negative effect of BIN2 on this transcriptional complex.

**Figure 7—figure supplement 1.**
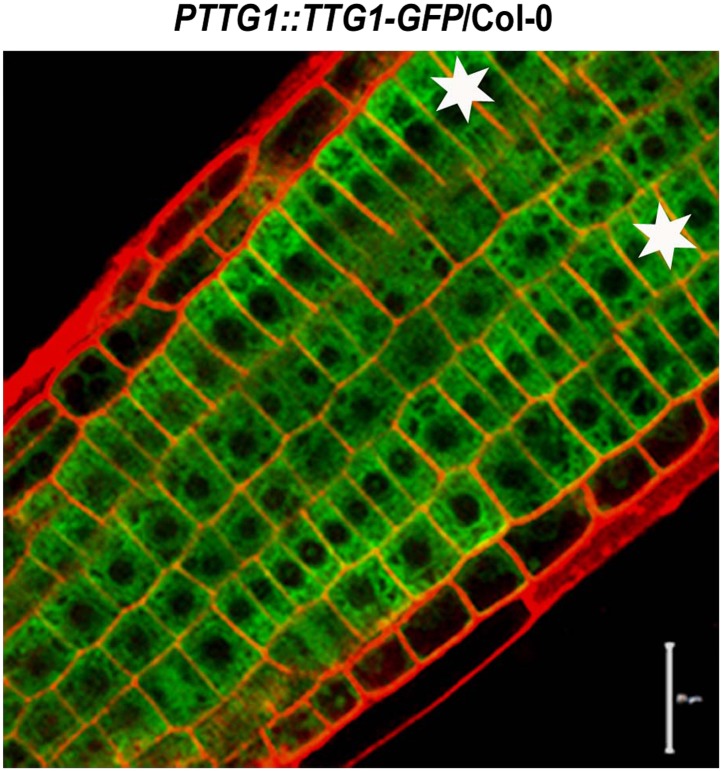
Subcellular localization of TTG1-GFP in Col-0 root epidermal cells. Stars indicate H cells. Scale bars, 20 μm. **DOI:** http://dx.doi.org/10.7554/eLife.02525.017

## Discussion

### BR signaling depends on GSK3-like kinases and the WER-EGL3-TTG1 complex to modulate root epidermal patterning

We provide several lines of evidence to strongly support an important role for BR signaling in directly regulating root hair cell fate. First, root hair patterning in the BR-biosynthetic and responsive mutants or in the wild type grown on eBL, bikinin, or BRZ, was dramatically altered, demonstrating that GSK3 kinases and/or their upstream components are mediating this regulation. Second, the expression pattern of the non-hair cell fate marker *PGL2::GUS* indicates that BR early signaling promotes N cell fate in the whole root epidermis, which is a reasonable explanation for the abnormal root hair patterning in the BR-related mutants: when BR signaling is enhanced, fewer root hairs are formed in the H position; when BR signaling is inhibited, more ectopic root hairs are produced in the N position. This finding supports the previous report that BRs positively regulate the expression of *WER* and *GL2* (Kuppusamy et al., 2009). Third, genetic analysis revealed that major components of the WER-EGL3-TTG1 or CPC-EGL3-TTG1 complex act downstream of BR signaling-mediated root epidermis patterning. Finally, BIN2 phosphorylation on EGL3 and TTG1 suggested that GSK3-like kinases directly regulate EGL3 movement and the transcription activity of the WER-EGL3-TTG1 complex to mediate root hair development.

### Phosphorylation of EGL3 regulates its intercellular movement and controls root epidermal cell fate

This study revealed a key mechanism in the regulation of intercellular communication of transcription factors by a hormonal signal to determine epidermal cell specification. Non-cell autonomous movement of some transcriptional factors is an important mechanism to regulate some developmental processes (Kurata et al., 2005b), and cytoplasmic localization of these mobile proteins may be required for their intercellular movement. For example, in maize shoot apical meristem, a mutation in its potential nuclear localization signal (NLS) of KNOTTED 1 abolished its intercellular movement (Vollbrecht et al., 1991; Lucas et al., 1995), and cytoplasmic localization of LEAFY (Schultz and Haughn, 1991), a transcriptional factor in floral identity, is also strongly correlated with its adjacent cell movement (Wu et al., 2003). Moreover, the movement of SHORT-ROOT, another mobile transcriptional factor in root radical patterning (Helariutta et al., 2000), was abolished when it was fused to a NLS, leading to its diminished cytoplasmic localization (Gallagher et al., 2004). However, it is largely unknown how the nuclear-cytoplasmic trafficking of transcription factors is regulated by internal cues to influence their intercellular movement. Although the intercellular movement of mobile factors in the WER/CPC-EGL3/GL3-TTG1 complex may determine root epidermis patterning (Savage et al., 2008), it is not clear how their movement is regulated by internal cues. Because *EGL3* mRNA was expressed only in H cells, the major nuclear localization of EGL3 protein in N cells indicated that, like GL3 and CPC, EGL3 can also move from H to N cells. In addition, we found that EGL3^T399A^ and EGL3^T209A/T213A^ with abolished phosphorylation sites were solely localized in the nucleus of H cells, indicating that the unphosphorylated EGL3 may not move between cells. Although we do not know how T209/T213 phosphorylation regulated EGL3 subcellular localization, the T399 phosphorylation likely affected a NLS, because T399 is located in a predicted non-canonical NLS (Figure 6—source data 1), which is found in many other plant bHLHs (Galstyan et al., 2012). Although the *EGL3*^*T399A*^*-GFP* plants showed normal root hair patterning, this can be explained by the close proximity of T399 to its bHLH domain, which may have affected its interaction with TTG1 and MYB (Zhang et al., 2003), leading to the nucleus-localized EGL3^T399A^ in H cells unable to induce *GL2* expression. However, EGL3^T209A/T213A^-GFP may still interact with TTG1 and have DNA-binding activity, because T209/T213 was located in the N-terminal region far away from the bHLH domain (Figure 6—figure supplement 1), which led to *GL2* expression in H cells and *EGL3*^*T209A/T213A*^*-GFP* plants growing fewer root hairs (Figure 6G; Table 1).

### GSK3-like kinases inhibit *GL2* expression through TTG1, which is required for the transcriptional activity of WER-EGL3-TTG1 complex

Besides the regulation of EGL3 nuclear-cytoplasmic trafficking, we also provided strong evidence to support GSK3-like kinases’ inhibition on the transcriptional activity of the WER-EGL3-TTG1 complex through TTG1. In *Nicotiana benthamiana* pavement cells, it was demonstrated that BIN2 has a negative role in WER-EGL3-TTG1 transcriptional activity, but has no effect on the activity of WER, EGL3, or both WER and EGL3. Although BIN2 phosphorylates EGL3, its failure to regulate WER-EGL3 transcriptional activity can be explained by a possible ubiquitous expression of *WER*, *EGL3*, and GSK3-like kinases in *Nicotiana benthamiana* leaves. Furthermore, it was reported that TTG1 interacts with EGL3 (Zhang et al., 2003), and TTG1 is necessary for the full functioning of other bHLH partners, such as GL3 and TRANSPARENT TESTA8 (Baudry et al., 2004; Zhao et al., 2008). Therefore, it is very likely that TTG1 phosphorylation by GSK3-like kinases may affect its regulation of EGL3 and the activity of the WER-EGL3-TTG1 complex.

### BR signaling promotes the N cell fate of root epidermis

Our data also support the suggestion that the N cell is a default cell type in root epidermis, and that H cell fate is produced due to inhibition of N cell fate by internal or external cues. First, we observed that *WER*, a positive regulator for *GL2* expression, is expressed in both N and H cells in the early root meristem (Figure 8—figure supplement 1), which is also supported by a previous report that *WER* exhibits uniform promoter activity in both N and H cells proximal to the initial cells (Savage et al., 2008). Second, in *Arabidopsis*, both *EGL3* and *GL3* are expressed in H cells, but their proteins move to adjacent cells to promote N cell fate (Bernhardt et al., 2005). If they stay in H cells with the ability to interact with WER and TTG1, the H cells may develop into N cells as shown in the *EGL3*^*T209A/T213A*^*-GFP* transgenic plants. Moreover, over-expression of *GL3* and *EGL3* promoted non-hair cell fate (Bernhardt et al., 2003). Third, TTG1 is localized in both N and H cells, and TTG1 and EGL3 may synergistically promote WER-EGL3 transcriptional activity and enhance N cell fate. Apparently, BR signaling can promote N cell fate in several ways. Besides the inhibition of BR signaling on EGL3 cell–cell movement and the promotion of TTG1 activity, BR signaling also promotes *WER* expression as *WER* up-regulation in *bin2-3 bil1 bil2* (Figure 8—figure supplement 1), which is consistent with the positive role of BRs in *WER* expression (Kuppusamy et al., 2009).

**Figure 8.**
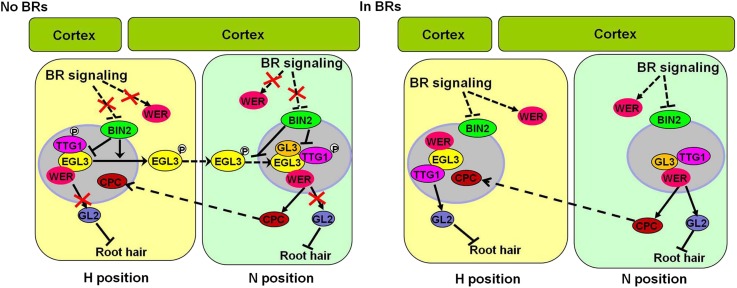
A proposed model to illustrate how BR signaling regulates root epidermal cell fate. Without BR early signaling, *WER* expression is reduced, and the activated GSK3-like kinases phosphorylate EGL3 and TTG1 in both H and N cells, leading to reduced formation and/or activity of the WER-EGL3/GL3-TTG1 complex, which inhibits *GL2* expression in some N cells. With enhanced BR early signaling, *WER* expression is enhanced in both H and N cells, and the GSK3-like kinases activity is inhibited, leading to reduced phosphorylation of EGL3 and TTG1 in both cell types. Thus, WER-EGL3-TTG1 and WER-GL3-TTG1 complexes with transcriptional activity are formed in H and N cells, respectively, to promote *GL2* expression and non-root hair cell fate. BR: brassinosteroid. **DOI:** http://dx.doi.org/10.7554/eLife.02525.018

Thus, we proposed a model to illustrate how BR signaling regulates WER-EGL3-TTG1 complex formation and activity to control root epidermal cell fate. As shown in Figure 8, without BRs, WER-GL3/EGl3-TTG1 complex formation and activity is inhibited in both N and H cells, as *WER* expression is reduced in both H and N cells, and the activated GSK3-like kinases phosphorylate EGL3 in H cells to promote its cytoplasmic localization in both H cells and N cells, both of which lead to less WER-GL3/EGL3-TTG1 complex formation in nuclei and suppression of *GL2* expression. The activity of some formed WER-bHLH-TTG1 complexes may be further inhibited by GSK3-like kinases phosphorylating TTG1. In contrast, enhanced BR early signaling inhibits GSK3-like kinases, leading to nuclear accumulation of the unphosphorylated EGL3 in H cells and normal function of unphosphorylated TTG1 in both cell types. Although CPC can move into H cells, due to enhanced *WER* expression and more efficient interaction of EGL3 with WER than with CPC (Song et al., 2011), more WER-EGL3-TTG1 complex is formed in H cells to promote *GL2* expression and determine N cell fate. In N cells, the nucleus-localized GL3 can interact with WER and TTG1 to promote *GL2* expression and maintain N cell fate. However, it remains to be investigated how *TTG1* and *WER* expression is regulated by BR signaling. It is also not clear how BR signaling coordinates with positional signals and other phytohormones to regulate root hair patterning.

**Figure 8—figure supplement 1.**
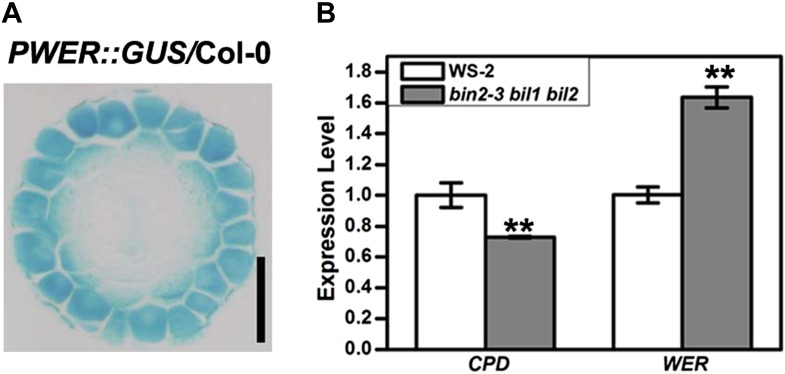
*WER* expression pattern in the root early meristem and its expression level in the *bin2-3 bil1 bil2* and wild type Col-0. (**A**) Transverse section from the root meristem of the *PWER::GUS* transgenic plant. Scale bar, 25 μm. (**B**) The *WER* expression level was enhanced in the *bin2-3 bil1 bil2* mutant. *CPD* (*CONSTITUTIVE PHOTOMORPHOGENIC DWARF*), a BR biosynthetic gene feedback inhibited by BR signaling, was used as a control. The expression level of *CPD* and *WER* in WS-2 was normalized to ‘1’, and a *U-BOX* gene (*At5g15400*) was used as an internal control. Error bars indicate SD. **p<0.01 with a two-tailed Student's *t* test. BR: brassinosteroid. **DOI:** http://dx.doi.org/10.7554/eLife.02525.019

## Materials and methods

### Plant materials and growth conditions

The seeds of the *wer-1* and *PGL2::GUS* lines were obtained from Dr John Schiefelbein (University of Michigan), the *bin2-3 bil1 bil2* seeds were obtained from Jianming Li (University of Michigan), and the *cpc-1* seeds (CS6399) were obtained from the Arabidopsis Biological Resource Center (Ohio State University). Combinations of the BR-related mutants with the root hair mutants or the *PGL2::GUS* line were generated by crossing and selected by GUS staining based on the mutant phenotype, or antibiotic selection marker analysis. For root hair observation, seeds were grown on 1/2 MS medium (pH 5.8) with 1% sucrose, chilled for 3 d at 4°C, and grown for 5 d at 23°C under long-day conditions (16 hr light/8 hr dark). *Nicotiana benthamiana* plants were grown at 28°C under long-day conditions (16 hr light/8 hr dark).

### Construction of double or multiple mutants

The double mutants or multiple mutants were derived from genetic crosses of the parental mutants (or transgenic lines). For generation of the *BRI1-OX wer-1* and *bil1 bil2 bil3 wer-1* double/multiple mutants, the *wer-1* was genotyped with its point mutation-derived cleaved amplified polymorphic sequence (CAPS) marker (Lee and Schiefelbein, 1999) (Supplementary file 1), the *bin2-3 bil1 bil2* was genotyped as described (Yan et al., 2009), and the *BRI1-OX* was selected by the antibiotic selection markers. For generation of *bri1-116 cpc-1* and *cpd cpc-1* double mutants, the *cpc-1* was identified by PCR and phenotypic analysis, and the *bri1-116* and the *cpd* were isolated by phenotype.

### Plasmid construction and recombined protein purification

For GST pull-down assays, *BIN2* was cloned into the *PET28a* vector, and *EGL3* was cloned into the *pGEX4T-1* vector. For in vitro kinase assays, *WER*, *TTG1*, *CPC*, and *EGL3* were cloned into the *pMAL-C2X* vector. His-fused BIN2 (BIN2-His), GST-fused EGL3 (EGL3-GST), and MBP-fused WER (WER-MBP), TTG1 (TTG1-MBP), CPC (CPC-MBP), and EGL3 (EGL3-MBP) were expressed in BL21 (DE3) pLySs strain and purified with either Ni-NTA agarose (Clontech, Mountain View, CA), glutathione resin (Genescript, Piscataway, NJ), or amylase resin (NEB, Ipswich, MA), respectively.

### Plant transformation and selection of transgenic plants

To generate plants expressing *GFP*-tagged *EGL3* or mutated *EGL3*^*T399A*^ and *EGL3*^*T209A/T213A*^, the various *EGL3* cDNAs were cloned in-frame with *GFP* into the *pCAMBIA2302* vector and driven by the *EGL3* promoter (2 kb upstream of the start codon), and the plants expressing *GFP*-tagged TTG1 were generated by cloning the *TTG1* cDNA in-frame with *GFP* into the *pCAMBIA2302* vector and driven by the *TTG1* promoter (2 kb upstream of the start codon). The constructs were transformed into *Agrobacterium tumefaciens GV3101* strains. All transgenic plants were generated by floral dip transformation. T_0_ seeds were harvested and screened by germinating on MS solid medium with antibiotic selection. For each transformation, at least five individual T_1_ transgenic lines were selected. Transgenic lines of *PEGL3::EGL3-GFP*, *PEGL3::EGL3*^*T399A*^*-GFP*, *PEGL3::EGL3*^*T209A/T213A*^-*GFP*, and *PTTG1::TTG1-GFP* with T_2_ or higher generations were used for further analysis.

### Microscopy and histochemical analysis

The root hair pattern of the 5-day-old seedlings was observed, and images at ×100 magnification were taken with a Leica MZ FLIII stereomicroscope (Leica Microsystems). The root hair density was counted as described (Galway et al., 1994; Jones et al., 2002) with some modifications. Any visible protrusion from the epidermal cell was regarded as a root hair, regardless of length. The number of root hairs was counted from one side of a 1 mm segment from the imitated differentiation region of the 5-day-old roots, and at least eight roots were measured for each stain. The hair cell length was measured along the longitudinal plane at ×100 magnification using the software Scion Image, and at least 10 root hair cells were measured for each root. The relative root number was calculated as root hair density × root hair cell length for each root as described (Wada et al., 1997).

The histochemical staining of 5-day-old roots harboring the *GUS* reporter was performed as described (Masucci et al., 1996). Transverse sections of root meristem were prepared as described (Ye et al., 2010) with modifications. The proportion of cells expressing or not expressing *PGL2::GUS* reporter in H cells or N cells was measured by examining sections at least from eight seedlings in each strain. For protein localization of EGL3-GFP and its mutated forms, the 5-day-old transgenic plants were examined by confocal microscope (Zeiss) after staining with 5 μg/ml propidium iodide (PI) (Sigma, St. Louis, MO) for 10 s at room temperature, and images were captured at 489 nm and 538 nm laser excitation and at 509 nm and 617 nm emission for GFP and PI staining. The pattern of epidermal cell types was determined as described (Lee and Schiefelbein, 2002).

### Yeast two-hybrid assay

For yeast two-hybrid assays, the full length cDNA of *BIN2* was cloned into vector *pEXP-AD502* (BIN2-AD) and used as a prey, and the full length cDNAs of *EGL3*, *WER*, *TTG1*, and *CPC* were cloned into the *pDBLeu* vector (EGL3-DB, WER-DB, TTG1-DB, CPC-DB), respectively, and used as a bait. The prey and bait plasmids were transformed into the yeast strains *AH109* and *Y187*, respectively. After yeast mating, the protein–protein interactions were tested on SD medium minus Leu, Trp, and His, and containing 2 mM 3-amino-1, 2, 4-triazole (3AT) (Sigma, St. Louis, MO).

### Transient expression assays in *Nicotiana benthamiana* leaves

To generate the vector system for BiFC analysis, the full length cDNAs of *EGL3*, *WER*, *TTG1*, and *CPC* were cloned into the *pXY104* vector (cYFP), respectively, to generate EGL3-cYFP, WER-cYFP, TTG1-cYFP, and CPC-cYFP constructs, and *BIN2* cDNA was cloned into the *pXY106* (nYFP) vector to generate BIN2-nYFP construct. For transient expression, *Agrobacterium* strains (GV3101) carrying the constructs for testing the specific interaction were transformed into 4–5-week-old *Nicotiana benthamiana* leaves as described previously (Walter et al., 2004). After infiltration for 4 d, the lower leaf epidermis cells were used for analyzing the fluorescence signal by confocal microscopy (Zeiss).

For dual-luciferase assays, cDNAs of the effectors *WER*, *EGL3*, and *TTG1*, and the regulator *bin2-1* were cloned with *Flag* tag into *pCAMBIA2302* driven by a 35S promoter. *GL2* promoter (2 kb upstream of the start codon) was cloned into the *pGreenII 0800-LUC* vector to be used as the reporter. The method of transient expression used was as previously described (Hellens et al., 2005).

### GST pull-down assays

The purified proteins, EGL3-GST, WER-GST, TTG1-GST, CPC-GST, and GST, were bound with 25 μl GST resin in binding buffer (10 mM phosphate buffer, pH 7.4, 140 mM NaCl, 3 mM KCl, 0.1% Triton X-100) for 2 hr at 4°C. After washing three times with the binding buffer, an equal amount of BIN2-His was added and rebound for 2 hr at 4°C. After boiling in SDS loading buffer for 5 min, the pull-down proteins were separated on 10% SDS–PAGE gels and detected by immunoblotting with anti-His antibody (Abmart, Shanghai, China).

### In vitro kinase assays and phosphorylation site identification

In vitro kinase assays were performed in 24 μl reaction buffer (20 mM Tris, pH 7.5, 10 mM MgCl_2_, 5 mM DTT) containing 20 μM ATP and 1 μl of 10 μCi [^32^P] γATP (PerkinElmer, Waltham, Massachusetts) and purified proteins. The reaction was carried out at 30°C for 1 hr and terminated by adding 6 μl of 5 × SDS loading buffer. After boiling for 3 min, proteins were separated on 10% SDS–PAGE. Gels were stained with Coomassie brilliant blue, and then dried and autoradiographed. For phosphorylation site identification, in vitro kinase assays were performed, and protein bands were excised to be used for mass spectrometry analysis.

### Site-directed mutagenesis of EGL3

The mutated forms of *EGL3* were generated by a PCR-based site-directed mutagenesis (Supplementary file 1).

## Additional files

Supplementary file 1. List of primers used in this study. **DOI:** http://dx.doi.org/10.7554/eLife.02525.020
