# Supplementary material for: Brassinosteroids control root epidermal cell fate via direct regulation of a MYB-bHLH-WD40 complex by GSK3-like kinases
Source: eLife. 2014 Apr 25;3:e02525. doi: 10.7554/eLife.02525 (PMC4005458; doi:10.7554/eLife.02525)
Supplement: Related texts

# Article commentary: Decision letter

**DOI:** 10.7554/eLife.02525.021

**Affiliations:** University of California, Berkeley and USDA Agricultural Research Service, United States

eLife posts the editorial decision letter and author response on a selection of the published articles (subject to the approval of the authors). An edited version of the letter sent to the authors after peer review is shown, indicating the substantive concerns or comments; minor concerns are not usually shown. Reviewers have the opportunity to discuss the decision before the letter is sent (see review process). Similarly, the author response typically shows only responses to the major concerns raised by the reviewers.

Thank you for sending your work entitled “Brassinosteroid signaling control root epidermal cell fate by GSK3-like kinases regulating WER-GL3/EGL3-TTG1 complex” for consideration at *eLife*. Your article has been favorably evaluated by a Senior editor (Detlef Weigel) and 2 reviewers, one of whom is a member of our Board of Reviewing Editors.

The following individuals responsible for the peer review of your submission have agreed to reveal their identity: Sheila McCormick (Reviewing editor) and John Schiefelbein (peer reviewer).

The Reviewing editor and the other reviewer discussed their comments before reaching this decision, and the Reviewing editor has assembled the following comments to help you prepare a revised submission.

This paper provides a significant advance in our understanding of the mechanism of brassinosteroid control of root hair cell pattern in Arabidopsis. While the first evidence for BR involvement in root hair patterning was published by Kuppusamy et al. (2009), this paper goes significantly beyond this early work, to include a broad array of experiments (including more BR-biosynthetic and signaling mutants, greater variety of BR inhibitors, and analysis of more epidermal patterning genes) and, most importantly, providing a clear mechanistic explanation (including evidence that the non-hair BR effect is mediated by GSK3-like kinases and evidence for interaction/phosphorylation of EGL3 and TTG1 by BIN2). Particularly exciting is that the work reveals an example of a core BR-signaling component targeting a non-BR transcription factor.

No additional experiments are required; the requested revisions are related to how the manuscript is written. It is not written with a general plant biologist in mind (i.e., someone who does not work on this system). There is a lot of jargon and thus the importance of the work may be obscure to some readers. The authors do not make it clear enough what is new about their work (compared to previous work), and they comment on previous work in an unconventional way. For example, the phrasing “this finding is also supported by a previous report”; more typically the phrasing would be “our results support the previously published result.” It is certainly fine to emphasize how your results are more substantial than those of Kuppusamy et al.

The quality of the English needs improvement. We suggest that you seek additional help from a native English speaker; the *eLife* editorial office can also give you advice or help to improve the writing.

# Reply: Author response

**DOI:** 10.7554/eLife.02525.022

*No additional experiments are required; the requested revisions are related to how the manuscript is written. It is not written with a general plant biologist in mind (i.e., someone who does not work on this system). There is a lot of jargon and thus the importance of the work may be obscure to some readers. The authors do not make it clear enough what is new about their work (compared to previous work), and they comment on previous work in an unconventional way. For example, the phrasing “this finding is also supported by a previous report”; more typically the phrasing would be “our results support the previously published result.” It is certainly fine to emphasize how your results are more substantial than those of Kuppusamy et al*.

*The quality of the English needs improvement. We suggest that you seek additional help from a native English speaker; the* eLife *editorial office can also give you advice or help to improve the writing*.

We have carefully checked the relevant phrases and revised them appropriately. A copy editor has also carefully checked the whole manuscript and provided great suggestions to improve the English writing in this version.
